# Design and implementation of automated notification systems and an electronic whiteboard for radiation therapy planning monitoring

**DOI:** 10.1002/acm2.14344

**Published:** 2024-04-14

**Authors:** Ping Yan, Jin Shen, Amar Basavatia, Madhur K. Garg, Wolfgang A. Tomé

**Affiliations:** ^1^ Radiation Oncology Montefiore Medical Center Bronx New York USA; ^2^ Radiation Oncology Albert Einstein College of Medicine Bronx New York USA

**Keywords:** automated process notification system, treatment planning, whiteboard

## Abstract

**Purpose:**

Radiotherapy (RT) treatment and treatment planning is a complex process prepared and delivered by a multidisciplinary team of specialists. Efficient communication and notification systems among different team members are therefore essential to ensure the safe, timely delivery of treatments to patients.

**Method:**

To address this issue, we developed and implemented automated notification systems and an electronic whiteboard to track every CT simulation, contouring task, the new‐start schedule, and physician's appointments and tasks, and notify team members of overdue and missing tasks and appointments. The electronic whiteboard was developed to have a straightforward view of current patients’ planning workflow and to help different team members coordinate with each other. The systems were implemented and have been used at our center to monitor the progress of treatment‐planning tasks for over 2 years.

**Results:**

The last‐minute plans were relatively reduced by about 40% in 2023 compared to 2021 and 2022 with a *p*‐value < 0.05. The overdue contouring tasks of more than 1 day decreased from 46.8% in 2019 and 33.6% in 2020 to 20%–26.4% in 2021–2023 with a *p*‐value < 0.05 after the implementation of the notification system. The rate of plans with 1–3 day planning time decreased by 20.31%, 39.32%, and 24.08% with a *p*‐value < 0.05 and the rate of plans with 1–3 day planning time due to the contouring task overdue more than 1 day decreased by 49.49%, 56.89%, and 46.52% with a *p*‐value < 0.05 after the implementation. The rate of outstanding appointments that are overdue by more than 7 days decreased by more than 5% with a *p*‐value < 0.05 following the implementation of the system.

**Conclusions:**

Our experience shows that this system requires minimal human intervention, improves the treatment planning workflow and process by reducing errors and delays in the treatment planning process, positively impacts on‐time treatment plan completion, and reduces the need for compressed or rushed treatment planning timelines.

## 1 INTRODUCTION

Radiation Therapy (RT) is a complex process encompassing task‐driven simulation, contouring, planning, therapy delivery, and follow‐up. Each step usually demands a series of manually created and completed time‐consuming tasks. These tasks are assigned to different patient care team members. At a minimum, this patient care team comprises a radiation oncologist, a medical physicist, a medical dosimetrist, a nurse, and a team of radiation therapists. Delayed or uncompleted tasks by any team member at any stage of the planning process can introduce unwanted delays in a patient's treatment start. Long waiting times have been associated with adverse clinical outcomes such as a higher risk of local recurrence,^1^ increased tumor progression,^2^ and prolonged psychological distress in patients.^3^

The radiation therapy treatment process starts with a patient's referral, followed by a consultation, after which a Computerized Tomography (CT) simulation is scheduled. In many cases, additional imaging studies such as Magnetic Resonance Imaging (MRI) or a Positron Emission Tomography (PET)‐CT are ordered and obtained to aid treatment target definition. Following the CT simulation, a treatment start date is set, the contouring tasks are assigned to the physicians by the therapists doing the CT simulation, the planning tasks are assigned to the planning team by the chief dosimetrist, and the plan checking tasks are assigned to the physics team by planning team when the plan is ready for checking. Timely completion of tasks ensures that treatment planning and treatment stay on track. The treatment plan generation can only start once the radiation oncologist has contoured the treatment target, organs at risk, and a treatment prescription has been created. Hence, any delays in completing these two tasks are a leading cause of delays in the treatment planning process and subsequent delays in the treatment start of patients. A recent survey identified the factors that expedite or prolong planning processes; chief among them is a physician's ability to provide contours on time and clear and timely communication between physicians and dosimetrists.^4^ Treatment planning team members usually take on the responsibility of reminding physicians if contouring is about to become overdue or is running late, calling a timely treatment start into question. This, however, involves significant effort that detracts from other tasks these team members need to complete to keep the treatment planning process moving along and can distract these team members from tasks at hand, leading to unneeded stress and possibly errors in the planning process. If treatment planning progress is monitored manually, the planner needs to check the treatment planning system for each patient to see whether the contouring process has been completed and a prescription has been entered. If not, keep reminding the physician to complete these tasks. If the planner forgets to check or is distracted by other tasks, the treatment planning workflow can be delayed. In addition to delays in treatment, this can lead to a reduction in treatment plan quality and safety of RT planning because of rushed completion of tasks to meet planning deadlines. Urgent last‐minute rush planning increases errors in Radiation Oncology.^5^ A smooth flow of information between care team members during the treatment planning process can directly impact the timeliness of clinical task completion.

As pointed out above, manually monitoring the treatment planning workflow is time‐consuming and can detract from other treatment planning tasks. A more efficient way of providing quality patient care and reducing manual labor is the adoption of a customized automated workflow management system that can (i) update the workflow in real‐time, (ii) allow team members to view and monitor the workflow of current patients easily, (iii) automatically send out notifications if tasks need to be completed, and (iv) provide an easier and more efficient automated way of communication.

To this end, several in‐house tracking systems or “whiteboards” have been developed and explored. These whiteboards allow users to visually track a patient's progress through the various stages of the planning process in real‐time and allow for real‐time updates on the planning status.^6^, ^7^, ^8^, ^9^ Gates and colleagues found that implementing a whiteboard reduced the proportion of needed next‐day physics Quality Assurance (QA)s by 27%.^7^ A custom electronic workflow management program was created and implemented by Medlever, Inc. Shailja and colleagues found that the program is effective in presenting supporting descriptive data across facility lines and multiple process steps.^8^ A whiteboard is a straightforward way to improve workflow efficiency in radiation oncology departments. There are two most used commercial Electronic Medical Record (EMR) Systems in Radiation Oncology. One is Aria Oncology Information System (Varian, Palo Alto) and the other is Mosaiq Radiation Oncology (Elekta, Sweden). Most whiteboards use either the tasks in Aria or the Quality Checklist (QCL) in Mosaiq. However, task tracking has limits. It depends on whether the tasks or QCLs are completed correctly and promptly. For example, in our clinic, tasks are sometimes completed but not marked as such in the oncology information system, derailing planning progress tracking. The CarePath in Aria is commercially available to track overdue tasks or appointments. However, it cannot provide efficient communication among different teams since the overdue tasks or appointments can only be seen by their owners or the same team. The uncompleted overdue tasks can also stop the activation of the rest of the tasks in the CarePath. If you want to know the CarePath of a patient, you need to open the patient's chart. The QCL also has similar issues. The overdue QCLs can only be seen by their owners or the same team. There is no communication among different teams.

Besides the tasks related to the planning progress, other tasks are also an essential part of radiation treatment workflow but have not been paid much attention to, such as “status check,” “follow up,” “peer review,” and the like. Varian's report system can run manually to get the list of uncompleted appointments. However, the reports include all the overdue appointments/tasks. They cannot be sent to a specific person or team. Currently, there is no other automatic tracking system to track these tasks. If these tasks are missed, there is no alert system to remind the responsible team member.

In this work, we report on a novel, in‐house electronic whiteboard and automated notification systems that automatically track tasks in real‐time and notify responsible team members whose tasks are overdue. The whiteboard displays a patient's treatment planning progress, and patients are ordered by treatment start time in descending order. The system has been implemented in our clinic for more than two years. We report on the performance of our system's clinical utilization by presenting workflow data before and after its implementation, showing its positive impact in aiding on‐time treatment plan completion and reducing the need for compressed or rushed treatment planning timelines.

## 2 METHODS

### 2.1 An electronic whiteboard system

An electronic whiteboard system was developed and deployed in our institution's Radiation Oncology Department. The whiteboard was generated using the Python Django web framework. Our center has the Aria Oncology Information System and Eclipse Treatment Planning System. The whiteboard system queries the Aria database every 60 min to update the displayed information. The whiteboard user can also reflash the web browser to update the planning information. To ensure the security of patient data, the website can only be accessed within the internal domain enclosed by the firewall. The whiteboard visually displays current new patient planning processes ordered by the port film starting date. The patient list reads the new treatment/port film appointments from the Aria database. The patient will not show up on the whiteboard if the new treatment/port film appointments have already passed the current time. The planning process starts with the CT simulation, followed by contour completion and planning status. To better review the planning process, the whiteboard displays the attending physician's and planner's names along with the machine and port date. Additional information, such as Rescan, SBRT, SRS, etc., is also in the “Note” column. Any overdue task will be displayed in different colors (green, yellow, red) depending on urgency.

As mentioned above, most current whiteboard systems use the “QCL” in Mosaiq or Aria's task/care path to track the planning process. However, the system will stop tracking if the task or the “QCL” is missing. Tracking replanning or boost planning using “QCL” is also tricky. The care path flow also depends on the task completion of the task's owner. If the previous task's owner forgets to complete the task or something stops the task completion, the rest of the tasks’ owners will not have their tasks as scheduled. For example, the planning task will not be triggered if the contouring task is not completed; the physics checking task will not be triggered if the planning task is not completed. We, therefore, check the treatment plan instead of using the “QCL” or the task that depends on manual assignment and completion and could introduce additional mistakes. A patient's treatment planning progress is highlighted in different colors if the planning tasks are either overdue (red), near the due date (yellow), or on track (green).

Our whiteboard includes 11 columns: “MRN (Medical Record Number),” “Name,” “Plan Status,” “MD (Medical Doctor),” “CT Date,” “MDIF (Medical Doctor input File ‐Rx and Contours Approved) Due,” “MDIF in,” “Planner,” “Machine,” “Port Date,” and “Note.”

The “CT Date” column shows when the CT images were acquired. Every CT simulation will trigger a new planning process. If the CT simulation appointment note states it is a rescan, this will also be shown in the whiteboard note column, and the original planning process tracking with the old CT will be closed out. The whiteboard always displays the date for the latest CT image set. Only the plans associated with this CT image set are tracked. Usually, there are several plans associated with one CT image set. It is difficult to tell which plan is correct for treatment using the computer. Therefore, we use plan names consistent with the latest physician clinical planning note to pick the right treatment plan. All plan names are generated using the following rules: Plan names should reflect the dose delivered in centigray (cGy). All plans submitted for physician approval shall be named “Planning Tumor Volume (PTV) + dose” for example, “PTV4500”. For plans treating multiple dose levels using a Simultaneous Integrated Boost (SIB) technique, the plan shall be named according to the highest dose treated and followed with “SIB” for example, “PTV6996_SIB”. For plan revisions, a version number shall be added to the plan name for example, “PTV4500_v2” (v3, v4 for subsequent replans as necessary). The clinical planning note contains disease‐specific planning information such as the dose prescription, motion management technique, desired treatment technique, and target and Organ At Risk (OAR) constraints. Although this method of selecting treatment plans does not have 100% specificity, we found it allows us to choose the correct plan with better than 95% accuracy. The 95% accuracy is based on estimating an average of one wrong treatment plan selected from about 20 new plans each day.

In our system, the contouring task is denoted by “MDIF.” The “MDIF DUE” column shows the physician's contouring task due date. It only shows the latest completed planning task indicated in the treatment plan. If there is no “MDIF” task in Aria, the column will display “none.” The “MDIF IN” column shows when the physician approved the contours. If the physician has not approved the contour after the due date, the column will turn red and display “overdue.” As we mentioned above, the tracking of the planning process is not based on the tasks. Whether or not the MDIF task is completed does not affect the system's judgment. The physician's approval of the Planning Tumor Volume (PTV) contour or other PTV‐related structures determines whether the contour is completed. For whole brain or breast plans, there sometimes are no PTV‐related structures. For these cases, physician approval of the “whole brain” or the breast contours will be used as the criterion.

The physician for each plan is read from the “Primary Oncologist” of the patient from the Aria database. The planner for each plan is the person who created the plan read from the Aria database. The planner's name will be blank if no plan is associated with the CT image. If there is no plan and the treatment will start in 3 days, “no planner” will be shown in the whiteboard “note” column to remind the chief dosimetrist to assign the planner for planning. The planning team can catch this mistake earlier and avoid further planning delays.

Six kinds of plan statuses are shown in the “Plan Status” column: “Plan not ready,” “Unapproved,” “Reviewed,” “Plan Approval,” “External Approval,” and “Treatment Approval.” If no plan can be found associated with the CT image set, it will show as “Plan not ready.” “Unapproved” means the planner is still working on the plan. “Reviewed” means the plan is undergoing pre‐plan checking by a medical physicist. The medical physicist and the physician are reviewing the plan concurrently. “Plan Approval” means the physician has approved the treatment plan, and the treatment planner and medical physicist are finalizing the plan. Some plans are done using different treatment planning systems, such as the Accuracy Precision for Tomotherapy and BrainLab for Stereotactic Radiosurgery (SRS). These plans are usually approved before being imported to the Eclipse Planning System and will be shown as “External Approval” on the whiteboard. When the whiteboard shows “Treatment Approval,” the plan is ready for treatment. If the port date is today, and the plans are not yet treatment approved, then “Plan Status” will be displayed in red. It will be shown in yellow if the port date is the following day.

For the boost plan, if the name of the boost plan is mentioned in the port film appointment note, the program will look for the plan with the same name. If not, it will look for the latest plan. The “note” column will also display the boost plan name.

### 2.2 Montefiore's radiation oncology planning progress monitoring system (MROPPMS)

We found email to be an excellent way to communicate. However, sending out reminder emails manually is time‐consuming, and this task is easily missed. Along with the whiteboard system, we created an in‐house notification system called MROPPMS using Microsoft C#.Net to ensure physicians delineate and approve the contours promptly so that planning can be completed on time and treatment delivered as scheduled. The MROPPMS takes in and tracks every CT simulation, each contouring task, along with the new‐start schedule from the Aria database. It will alert the planning and administration teams if the port or first‐day treatment is within 3 days and no contouring task is issued. It will notify the physician to complete and approve the contours if they are not approved and the contouring task is overdue. If the port date is the next day and the contouring task is overdue, it will be displayed in red.

**FIGURE 1:**
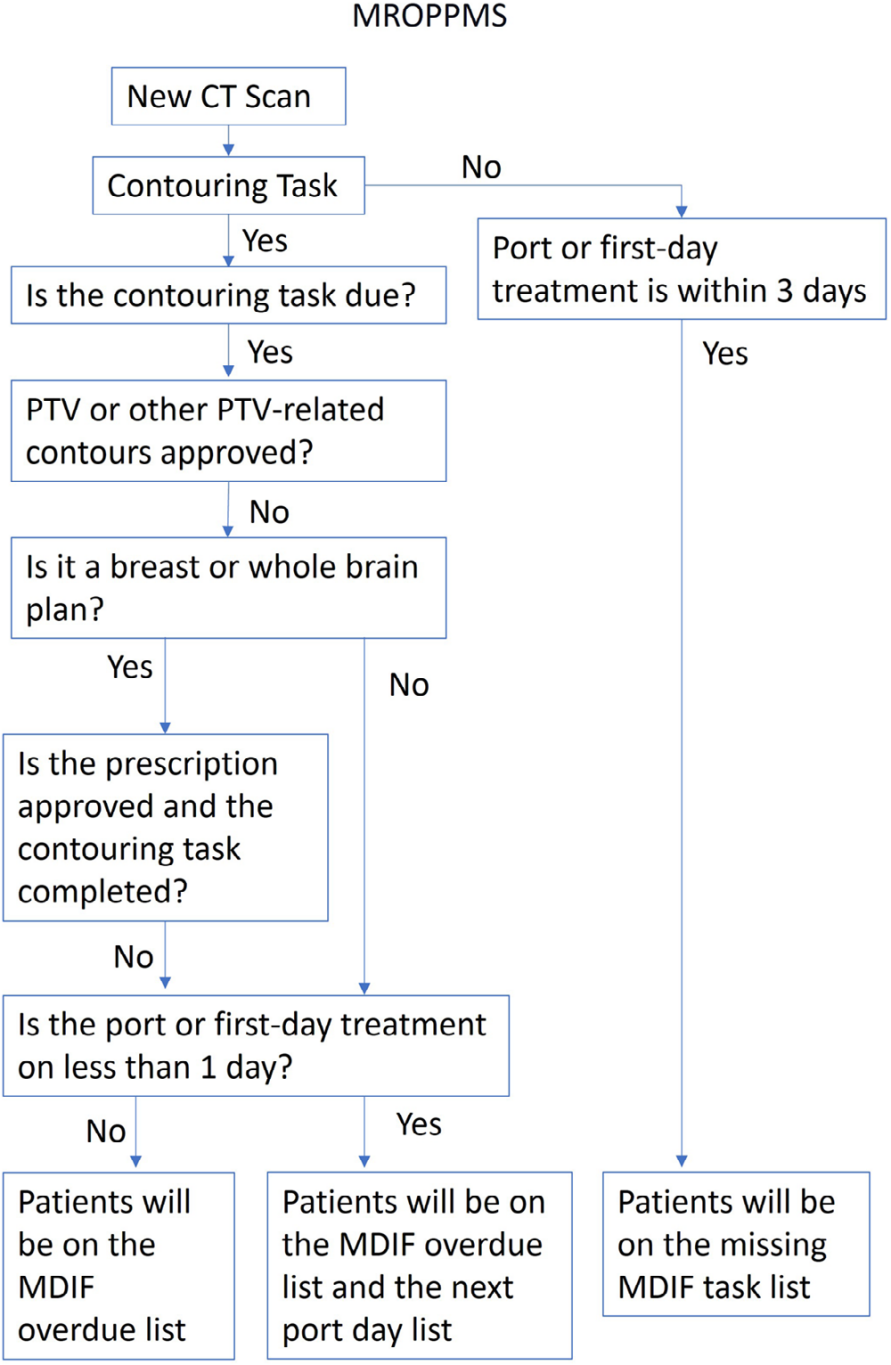
The logical flow diagram for MROPPMS.

The MROPPMS checks the approvals of the PTV or other PTV‐related contours in the Eclipse Treatment System for the most recent CT simulation instead of the completion of the MDIF task. However, there are sometimes no PTV or other PTV‐related contours for breast or whole brain plans. For these exceptional cases, the physician's prescription approval and the MDIF task's completion will determine whether the contour is completed. The logical flow diagram for MROPPMS is shown in Figure 1.

### 2.3 Montefiore's radiation oncology open appointment monitoring system (MROOAMS)

Besides the contouring tasks, other tasks and appointments such as the “consult,” “status check,” “Follow‐up,” and so on are also essential for the workflow in the Radiation Oncology Department. We created an in‐house system called MROOAMS using Microsoft C# Net to check and remind responsible team members if any appointment or task needs to be completed. The software queries the Aria database for all the tasks due in the past week. Emails are sent to each physician with uncompleted appointments or tasks every Monday. The administrative team creates the tasks and appointments in the Aria system when the treatments are scheduled. The physicians need to complete and bill for each task after they finish the task. If the physician forgets to complete the appointments or the tasks, the department will lose the billing for the uncompleted tasks. Sometimes, the patient does not show up or misses an appointment. If the physician fails to tell the administration team or the nurse, no one will reschedule a new appointment for the patient. Before this email notification system was implemented in our department, the administrative team ran the Varian report once every month and reported in our department's routine QA meeting. This automatic email notification system is a better way to catch these cases quickly.

## 3 RESULTS AND DISCUSSION

### 3.1 Whiteboard implementation

**FIGURE 2:**
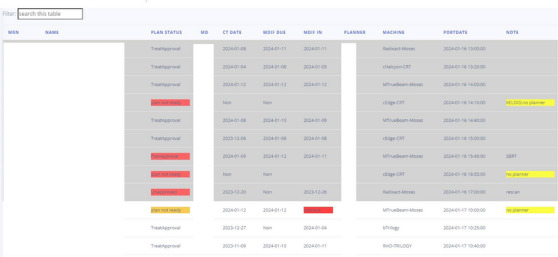
A screenshot of the whiteboard implemented in our department.

The whiteboard was implemented in 2022 in our department. Our department has three sites. Two of the sites each have two machines. One site has one machine. We have 16 physicians, 11 dosimetrists, and 12 physicists. The main page of the whiteboard is shown in Figure 2. It shows at which stage of the planning process a patient is at. By default, patients are listed in the order of their starting port date. However, one can sort the list differently, using a filter box to search patients by name, machine, physician, planner, etc.

All patients undergoing treatment planning and the current stage at which they are in the planning process are displayed on the whiteboard. Therefore, it is straightforward to determine whether a given plan is ready for the treatment or not. Delayed processes are highlighted in different colors, making it easy to see whether the plan is delayed, and at which stage it is. We set the table to be automatically updated every 60 min to avoid too frequent inquiries about the database but keep the data up to date. In addition, the user can refresh the table to update all the changes in the database at any time.

At the beginning of the implementation, we checked the whiteboard with Treatment Planning System Eclipse and Medical Record System Aria every day. If any information was inconsistent among them, an investigation was carried out, and the problems identified were corrected. This process lasted for approximately 3 months. After that, if users find any inconsistent information or have any suggestions, they will report them to the developer of the whiteboard. If the suggestions are approved, the whiteboard will be updated after the modification.

### 3.2 Planning process analysis

**FIGURE 3:**
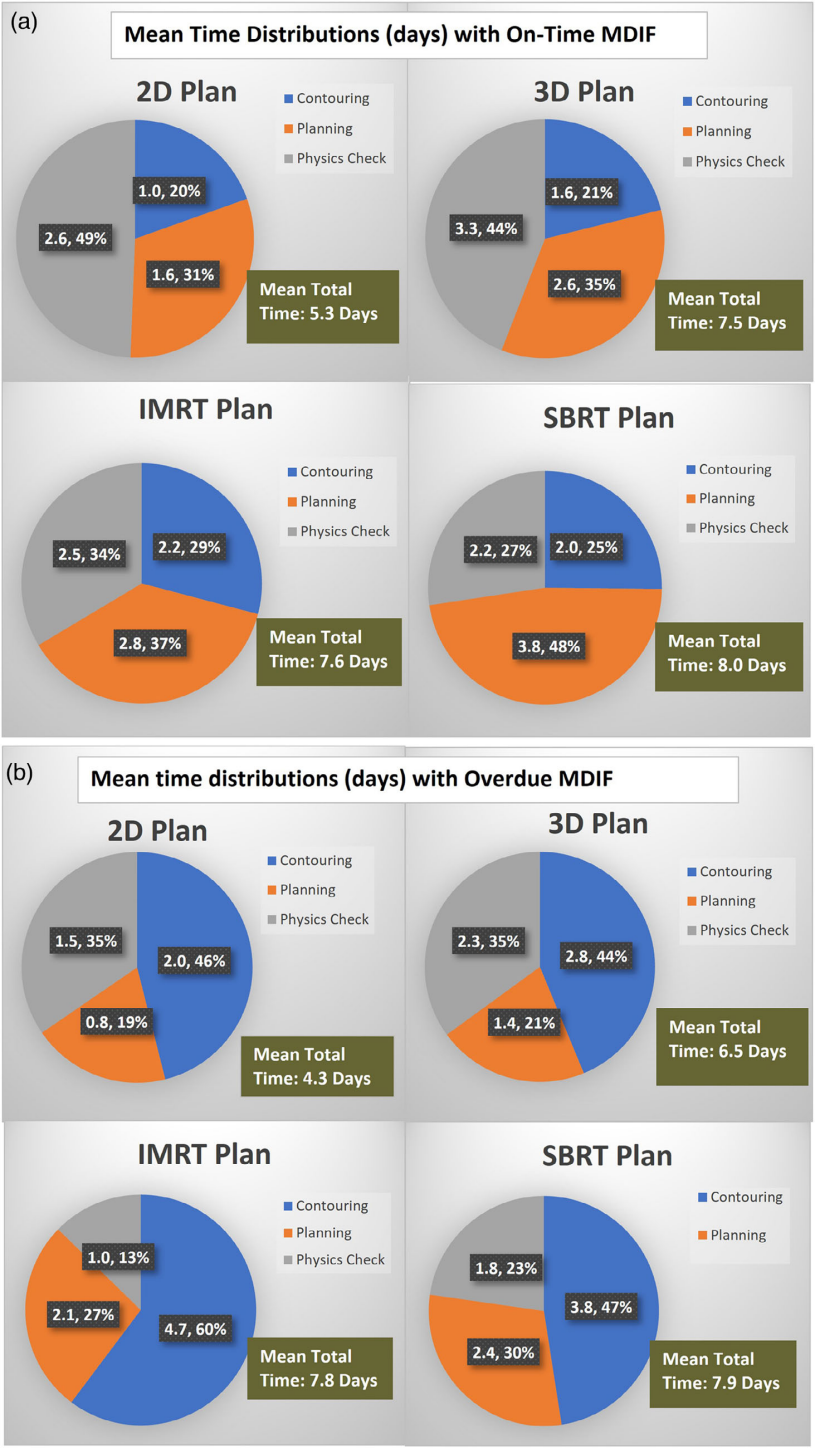
(a) Mean time distributions (days) with on‐time MDIF; (b) Mean time distributions (days) with overdue MDIF.

Data used for analysis spanned the period from January 2021 to August 2023 and includes 2497 plans treated at our institution. The percentage of 2D, 3D, IMRT, and SBRT plans were 10.53%, 41.57%, 30.96%, and 16.94%, respectively. Figure 3(a),(b) shows the mean times for three planning process stages: “Contouring,” “Planning,” and “Physics Check,” tracked in our Oncology Information System (OIS) since 2021. The percentage distribution of median times for four types of plans (2D, 3D, IMRT, and SBRT) with on‐time MDIF and overdue MDIF are shown from the top left to the bottom right. The overdue MDIF did not affect the total time of the planning process. The mean total times of the planning process are about 4−5 days for the 2D plan, 6−8 days for the 3D plan, and 8 days for the IMRT/SBRT plans (cf. Figure 3(a),(b)). This turnaround time is similar to that reported in.^8^ Hence, overdue MDIFs reduce the time available for treatment planning. For 2D plans, there is less than 1 day for planning (cf. Figure 3(b)). This means the plan is done on the same day the contour is approved. For other plan types, if the MDIF is on time, the percentage of planning time is generally more than 30% of the total planning process (cf. Figure 3(a)). If the MDIF is overdue, the rate of planning time is less than 30% of the entire planning process (cf. Figure 3(b)). For the IMRT and SBRT plans, if the MDIF is on time, there are about 2 days for physics checking. It includes chart checking, IMRT QA finalizing the plan report, etc. If the MDIF is overdue, there is about 1 day for physics checking. Shortening the time for planning and physics checks can result in a low‐quality plan and overlooked planning mistakes.

The last‐minute plan is defined as the plan checked and finalized on the day of treatment. Table 1 shows the percentage of last‐minute plans completed on the day of the scheduled appointment for 2021−2023. The January to March 2022 data were excluded from 2022 and added to 2021 because we implemented the whiteboard in April 2022. Plans with a total planning process time from simulation to treatment start of less than 4 days, since they are mostly emergency plans, were excluded from the analysis. There are many reasons for last‐minute plans, but the two main reasons are overdue contours and planning errors. The last‐minute plan with errors is defined as a plan with more than 3 days of planning, but the task for the physics check is assigned on the port/first treatment day. These plans could be accidentally missed by the planners, or the plans with major mistakes must be replanned. Following the implementation of our whiteboard system, the total for last‐minute plans decreased from 4.29% (2021), 4.95% (2022) to 2.85% (2023) with a *p*‐value of 0.032, while last‐minute plans due to overdue MDIF did not have significant change, and the percentage of last‐minute plans due to planning errors decreased from 2.09%(2021), 2.18%(2022) to 1.03%(2023) with a *p*‐value of 0.0552 (cf. Table 1). There are not many changes from 2021 to 2022. This means that it takes time for people to use the whiteboard to monitor the planning processes. The ratios of last‐minute plans due to delayed MDIF to all last‐minute plans are 46.15% (2021), 56.89% (2022), and 68.12% (2023) (cf. Table 1). This indicates that delayed contouring is the main driver for needed last‐minute plans. On the other hand, last‐minute plans and last‐minute plans due to planning errors showed a relative reduction of about 40% and 50% in 2023 compared to 2021 and 2022, indicating that the whiteboard helped to reduce the last‐minute plans, especially the last‐minute plans with errors.

**TABLE 1:** Percentage of last‐minute plans for 2021−2023, including the total last‐minute plans, the last‐minute plan due to the overdue MDIF, and the last‐minute plan with planning day > 3 days.

Year	Last‐minute plan (all)	Last‐minute plan (overdue MDIF)/ratio	Last‐minute plan (>3 planning days)/ratio
2021	4.29%	1.98%/46.15%	2.09%/48.63%
2022^a^	4.95%	2.82%/56.89%	2.18%/44%
2023	2.85%	1.94%/68.12%	1.03%/36%
*p*‐value	0.0320	0.2700	0.0552

^a^ Data from January to March were excluded from 2022 and added into 2021.

### 3.3 MROPPMS implementation

Before the MROPPMS implementation in November 2020, planning team members manually monitored the progress of the treatment planning process. A planning team member checked the contouring process in the OIS and sent daily reminder emails. Starting in November 2020, following MROPPMS implementation, emails were automatically sent to physicians whose tasks were overdue at the end of the working day. Only the physicians whose MDIF is overdue will receive the email. The planning team and administrative, physics, and physician team leaders will also be notified. At the beginning of the implementation, the developer of the MROPPMS checked the list manually with the planning team. This process was about 2−3 months.

**FIGURE 4:**
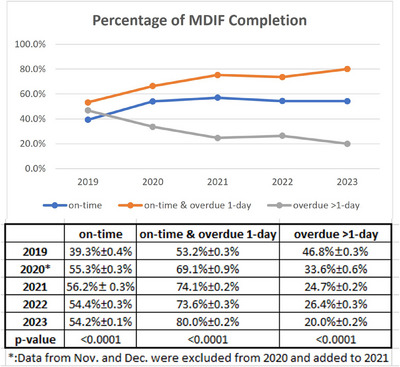
Percentages of MDIF tasks completed on time, on‐time & overdue 1 day, and overdue >1 day.

Data used for the analysis of the MROPPMS spanned the period from 2019 to 2023. Figure 4 shows our analysis of overdue contour tasks throughout monthly data from 2019 to 2023 regarding MDIF tasks being on time, on‐time and overdue 1 day, and overdue >1 day. Since the MROPPMS was implemented in November 2020, the November and December 2020 data were excluded from 2020 and added to 2021. The data were analyzed using the ANOVA test. Although the rates of on‐time MDIF differences in 2020−2023 are similar, the percentages of MDIF tasks on time & overdue 1 day were increased by about 5%−10% (*p*‐value < 0.05) after the implementation of MROPPMS, indicating that more physicians approved their contours within 1 or 2 days of receiving the notification email.

**FIGURE 5:**
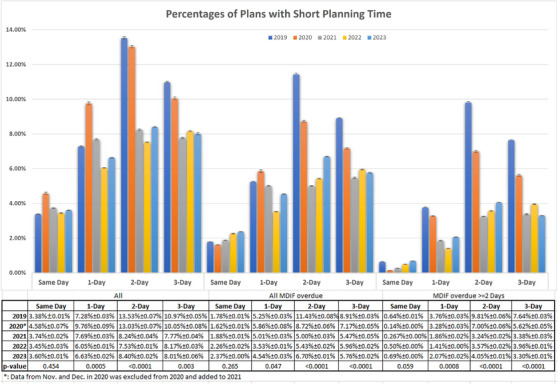
Percentages of plans with short planning time for all, plans with MDIF overdue and plans with MDIF overdue ≥2 days from 2019 to 2023.

Due to the delays in contouring, the remaining planning time is often compressed to less than the scheduled 3 days, which is the minimum planning time for our department except for emergency planning. Data used for analysis were monthly data from 2019 to 2023. The data from November and December 2020 were added to 2021. Figure 5 depicts the percentage of plans with short planning time, with less than 3 days of planning, plans with short planning time due to MDIF overdue, and MDIF overdue more than 1 day. We found that the *p*‐value of planning on the same day is more than 0.05. It means that the comparisons for same‐day planning are not statistically significant. The rates of plans with 2‐day and 3‐day planning time decreased by about 3%−5% with *p*‐value < 0.05, a relative average decrease of 24%−39% after the implementation of MROPPMS (Table 2). The rate of plans with short planning time (1–3 days) due to MDIF overdue more than 1 day decreased from 3.53%(1‐day), 8.4%(2‐day), 6.63%(3‐day) to 1.78%(1‐day), 3.62%(2‐day), and 3.54%(3‐day) after the implementation of MROPPMS, a relative average decrease about 50% as shown in Table 2. This means that more MDIF tasks were completed within 1 day of notification after implementing the MROPPMS, thus reducing the number of plans with shortened planning time and that these gains are durable and can be maintained.

**TABLE 2:** Percentages and relative rates of plans with short planning time, plans with short planning time due to MDIF overdue and MDIF overdue ≥ 2 days before (1/2019–10/2020) and after (11/2020–12/2023) the implementation of MROPPMS.

	All plans				Plans with MDIF overdue				Plans with MDIF overdue ≥2 Days			
Implementation	Same day	1‐day	2‐day	3‐day	Same day	1‐day	2‐day	3‐day	Same day	1‐day	2‐day	3‐day
**Before**	3.98%	8.52%	13.28%	10.51%	1.70%	5.55%	10.07%	8.04%	0.39%	3.52%	8.40%	6.63%
**After**	3.60%	6.79%	8.06%	7.98%	2.17%	4.36%	5.71%	5.73%	0.49%	1.78%	3.62%	3.54%
**Relative rate**	9.62%	20.31%	39.32%	24.08%	−27.74%	21.45%	43.33%	28.73%	−25.12%	49.49%	56.89%	46.52%

### 3.4 MROOAMS implementation

**FIGURE 6:**
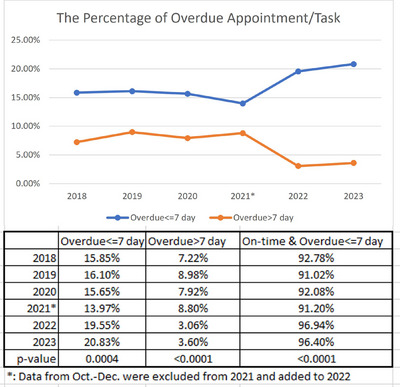
Percentage of appointments overdue from 2018 to 2023.

The MROOAMS was implemented in October 2021. It runs on Monday every week, and emails are automatically sent to physicians whose appointments are not completed on time. We analyzed the patient data for the 6 years from 2018 to 2023. While the percentage of appointments that are overdue less than 7 days increased by about 5% points after the OAMS implementation, the rate of outstanding appointments that are overdue more than 7 days decreased by more than 5% points, the total appointments that are on time and overdue less than 7 days increased by about 5% with *p*‐values less than 0.05 following the implementation of MROOAMS (cf. Figure 6). This indicates that the physicians completed most appointments after receiving the notification emails. We found that MROOAMS is also an excellent way to communicate between administrative team and physician teams.

## 4 CONCLUSION

We developed and successfully implemented whiteboard and email notification systems in our department. Implementing the whiteboard and email notification systems requires minimal user intervention and, as our data shows, can improve the treatment planning workflow of radiotherapy and enhance communication among team members. The software queries the database directly. It can be adapted to a different system by changing the query as appropriate for its database structure. If the Treatment Planning database is different from the Medical Record System database, the software will need to be modified to query both the Treatment Planning database and the Medical Record System database. The planning process monitoring system is independent of the completion of tasks. It tracks the plan status directly and does not rely on the “Care Path” of Aria or the “QCL” of Mosaiq. The system can improve the efficiency and accuracy of the workflow. Most importantly, the system allows each group adequate time to safely create and deliver patient plans. Ongoing work focuses on expanding our email notification system to other groups of the care team, such as the planning and physics team, for overdue tasks to further shorten and streamline timelines to care.

## AUTHOR CONTRIBUTIONS

Ping Yan, Amar Basavatia, Madhur K. Garg, and Wolfgang A. Tomé contributed to the software conception and design; Ping Yan contributed to the software development and implementation, analysis, and interpretation of results; Ping Yan and Jin Shen contributed to the Data acquisition.

## CONFLICT OF INTEREST STATEMENT

The authors declare no conflicts of interest.
